# Origins in the USA in the 1980s of the warning that smokeless tobacco is not a safe alternative to cigarettes: a historical, documents-based assessment with implications for comparative warnings on less harmful tobacco/nicotine products

**DOI:** 10.1186/s12954-018-0228-8

**Published:** 2018-04-16

**Authors:** Lynn T. Kozlowski

**Affiliations:** Department of Community Health and Health Behavior, School of Public Health and Health Professions, University at Buffalo, State University of New York, 323 Kimball Tower, 3435 Main Street, Buffalo, NY 14214-8028 USA

**Keywords:** Smokeless tobacco, Cigarettes, Vape, E-cigarettes, Health information, Public health ethics, Warnings, Policy, Harm reduction, Risk communication

## Abstract

**Background:**

Before the 1980s in the USA, smokeless tobacco carried no health warnings, was not judged to cause disease, and was a declining practice. In 1986, the federal government passed legislation requiring rotating warnings on “mouth cancer,” “gum disease and tooth loss,” and “This product is not a safe alternative to cigarettes.” This paper explores the history of the establishment of these warnings with emphasis on the ‘not a safe alternative’ warning and the bases for claiming that smokeless was ‘not safe’ (absolute harm) versus ‘not safer than cigarettes’ (relative harm).

**Methods:**

Results of searches of Truth Tobacco Industry Document archives and transcripts of legislative hearings were analyzed. Critical assessments were made of the evidence-base.

**Results:**

New evidence of oral cancer causation emerged along with a much-publicized case of a teenager dying of oral cancer. Public health concerns also arose over a widespread, successful marketing campaign implying smokeless was a safe alternative to cigarettes. Industry wanted pre-emptive federal warnings, to prevent a diversity of pending state warnings. To avoid an addiction warning, the industry accepted a compromise ‘not a safe alternative’ warning, which had not been initially proposed and which the cigarette industry may have sought in order to constrain the smokeless tobacco industry. The evidence presented supported smokeless only as ‘not safe’ and not ‘as harmful as cigarette smoking.’

**Conclusions:**

The comparative warning was a compromise to prevent an addiction warning and consistent with the preferences of cigarette companies. Prior surveys indicated that the public generally did not view smokeless tobacco as harmless, but they did generally report smokeless as less harmful than cigarettes despite expert interpretations to the contrary. As would not have been appreciated by public health supporters at the outset, subsequent research has shown that the ‘not a safe alternative’ message is misinterpreted by consumers to indicate that smokeless is ‘not safer’ than cigarettes—which was not established and has been disconfirmed by subsequent assessments of that question. Though many countries have banned smokeless tobacco (but not cigarettes), where smokeless is legally available accurate information on the nature of harms and differential harms needs to be developed.

## Background

Before 1981 in the USA, smokeless tobacco (SLT) carried no health warnings, had not been officially determined to cause disease, and was an unpopular and declining practice [1]. The 1964 Surgeon-General Report [2] found “...no useful mortality statistics in those who chew, snuff, or “dip” tobacco” (p74). The 1979 Surgeon-General Report [3] concluded: “Snuff and chewing tobacco have not been found to increase mortality (either overall or cause-specific) in the United States” (Chapter 1, p. 29). For oral cancer and leukoplakia, it concluded: “...there does seem to be an association between tobacco chewing and leukoplakia and oral cancer in Asia, but it is not clear that the same risk holds true in the United States due to a difference in the tobacco being chewed and to differences in the nutritional status and other characteristics of the population.” (Chapter 13, pp. 41–42). SLT was advertised on radio and television; cigarettes had not been since 1971. There had been a warning and widespread publicity on the health risks of smoking since the late 1960s.

In 1985, the US Congress was strengthening the required warnings for cigarettes, and in 1986, the first warnings would be required on SLT [4]. These exact cigarette warnings have continued for the past three decades (see Table 1). For SLT, the only major change has been the addition of a warning about addiction (see Table 1 for the 1986 warnings). This historical analysis will look most closely at the creation of the warning, “This product is not a safe alternative to cigarettes,” (NSAC) which was not among the initially proposed warnings and appeared as a compromise late in the process. Subsequently, a NSAC warning has been (a) among warnings recommended internationally by the World Health Organization [5], (b) the object of more recent research on consumer interpretation of the message [6, 7], and (c) considered for potential use for other types of less harmful tobacco/nicotine products such as vaping [8].

**Table 1:** Required warnings for cigarettes (1985) and for smokeless (1986), including proposed and compromise warnings

Cigarettes: ‘surgeon general’s warning’	Smokeless tobacco: ‘warning’
Smoking causes lung cancer, heart disease, emphysema, and may complicate pregnancy	This product may cause mouth cancer
Quitting smoking now greatly reduces serious risks to your health	This product may cause gum disease and tooth loss
Smoking by pregnant women may result in fetal injury, premature birth, and low birth weight	Proposed: this product contains nicotine and is addictive
Cigarette smoke contains carbon monoxide	Compromise: this product is not a safe alternative to cigarettes

It has been argued that the NSAC message under-informs consumers by not also informing them accurately that smokeless is much less harmful than cigarettes [9]. Recent research [6, 7] shows that individuals can also be misinformed by the NSAC message by interpreting it as conveying that SLT is not a *safer* alternative to cigarettes (i.e., just as harmful as cigarettes). A particular interest was to explore the extent to which the evidence available to justify the first SLT warnings indicated that SLT was (1) not safe or harmless in its own right or (2) not safer or less harmful than cigarettes. The first is a question of absolute harm of SLT, compared to non-use of SLT. The second is a question of relative harm (harm reduction), compared to cigarettes. Tobacco industry document archives were used to provide a sense of the historical context both behind the scenes as well as in the public arena.

## Methods

Systematic searches on the main questions were done using the “Truth Tobacco Industry Documents” web archive at the University of California, San Francisco Library. These questions focused on the knowledge of SLT harms, the creation of SLT warnings, and the evidence and concern that SLT was not safe or not safer than cigarettes. The archive provided access not only to internal industry documents, but also to media reports, commentaries, anti-tobacco documents, and legislative materials that the companies found relevant. These non-industry materials were also used to help understand what was going on external to the industry. The time period from 1964 to 1990 was used. Searches were done on all the key legislators and their aides as well as industry and health group participants. Legislative bills were searched. Search terms included smokeless, snuff, chew, “chewing tobacco,” “not safe,” “not a safe alternative,” harmless, warning, labels, safer. Special attention was paid to testimony at hearings related to the legislation. Research from the time that was part of the record was assessed critically.

## Results

Once the many duplicate documents were removed, over 400 documents were consulted, including internal industry documents as well as external documents that were in the industry files.

### Smokeless tobacco ‘discovered’ to cause cancer

#### 1981—National Cancer Institute finds snuff causes oral cancer

In March 1981, “Snuff dipping and oral cancer among women in the southern United States,” a study of 255 cases and 502 controls, [10] was featured in the *New England Journal of Medicine* by Winn and others from the National Cancer Institute (NCI). The abstract reported “The relative risk associated with snuff dipping among white nonsmokers was 4.2 (95% confidence limits, 2.6 to 5.7), and among chronic users this risk approached 50-fold for cancers of the gum and buccal mucosa...” (p. 745). The authors considered their study conclusive and “...the first to show a definite link” [11]. The results were widely publicized, including in January 1983 on national television [12]. The NCI issued a statement entitled, “In Answer to Your Questions About Smokeless Tobacco,” [13]. It treated the snuff-dipping study as if it were conclusive evidence against SLT.

Many continue to accept the Winn et al. study as applicable to the dominant form of SLT (moist snuff) (see, [14]), but it actually mainly concerned a different product—dry snuff. Winn responded to a question at a conference in September 1985 that the women studied used “almost exclusively dry snuff” (p 325). Dry snuff was much less popular than moist snuff and has declined even further in use [15]. Unlike for moist snuff, ‘snuff-dipping’ involved chewing a twig to make a brush that was ‘dipped’ into dry snuff and placed in the mouth [16]. Dry snuff is different chemically from moist snuff and likely more carcinogenic [17]. None of these limitations on the generalizability of these findings to moist snuff were discussed in legislative assessments. Further critiques of the NCI study are available (e.g., [17])

#### 1984–1986—the Sean Marsee case increases public fears over smokeless as a cause of cancer

The tragic death of Sean Marsee at age 19 from tongue cancer was blamed on SLT and became a *cause célèbre* for anti-SLT supporters [4]. His mother brought a lawsuit unsuccessfully against the manufacturer. The lawsuit and his death were widely publicized in major national media starting late in 1984, in hearings, and on major national television shows (e.g., [18]).

### Skoal Bandits® develops new markets as an alternative to cigarettes

In July 1983, the United States Tobacco Company (UST) announced its largest marketing campaign ever in the New York area for Skoal Bandits® which was wintergreen-flavored moist snuff in individual serving sachets [19]. Skoal Bandits® were promoted as an alternative to smoking, “Take a pouch instead of a puff.” A report in Advertising Age [20] was entitled “Bandits steal a bite from cigarettes” and a picture caption noted “Ads seek to win over urban smokers trying to kick the habit.” Along with TV spots on cable and commercial TV, there were radio and newspaper spots, as well as special events featuring the Skoal Bandits® race car team and popular athletes.

The promotion of Skoal Bandits® triggered a response from the President of Lorillard Tobacco Company to the Chairman of the Board of UST: “I told him that my company and I were concerned with the ‘Ad Age’ article showing his new campaign on ‘Skoal Bandits’ (‘Take a pouch instead of a puff’) and that we felt that he should be able to sell his product (in fact, he had been able to grow beautifully) without denigrating cigarettes” [21].

From 1978 to 1985, sales for moist snuff increased by 55%; by 1985 there were 13 million users, 3 million below the age of 21 [1]. Moist snuff was the only growing segment of the tobacco market [22]. SLT use by youth and adults was increasing in all parts of the country [1, 22]. A television commercial appeared as part of Winter Olympics coverage, with testimonials of converts from cigarettes [22].

### The public health community responds that smokeless is ‘not a safe alternative to cigarettes’

In February 1984, a petition to the Federal Trade Commission from Ralph Nader’s consumer-focused organization, The Public Citizen Health Research Group (PCHRG), argued that warnings were needed for SLT [23]. The Winn et al. study was highlighted and some students were interviewed. The petition argued that without warnings consumers will likely believe SLT is safe. The cover letter referred to the $30 million campaign for Skoal Bandits® and noted: “...the campaign slogan, ‘Take a pouch instead of a puff,’ implies that Skoal Bandits are a safe alternative to cigarette smoking. They are not.”

In the spring of 1984, the New York State Attorney General formally complained about Skoal Bandits® advertising [24]. He charged that the warning on cigarettes, coupled with the “Take a pouch instead of a puff” slogan, implied that smokeless was safe, *unless it also had a warning that it was not safe*. By May, UST had decided to not fight the issue and agreed to stop such advertising [24]. When it was learned that a promotional giveaway of tote bags was scheduled for the Yankee baseball Stadium after the advertising was set to end, a supplemental deal was arranged. It allowed distribution of the bags with the addition of a sticker that said: “OUR SLOGAN ‘TAKE A POUCH INSTEAD OF A PUFF’ DOES NOT IMPLY THAT USE OF SKOAL BANDITS IS A SAFE ALTERNATIVE TO SMOKING: U.S. Tobacco, Makers of SKOAL BANDITS” [25].

#### States seek warning labels

The Massachusetts Department of Dental Health led regulatory efforts against SLT [4] and organized hearings in February 1985 [26, 27]. Experts, parents, coaches, and student athletes testified that SLT was thought to be a safe alternative to smoking. A warning label was proposed and passed into Massachusetts law by July 1985, requiring the label in December 1985: “Warning: Use of snuff can be addictive and can cause mouth cancer and other mouth disorders.” By January 1986, 26 states passed legislation requiring warning labels, many noting ‘nicotine’ and ‘addiction’ in the warning, and none initially including a ‘not a safe alternative to cigarettes’ (NSAC) warning [28]. These state actions pressured the federal government to act and the SLT industry (the Smokeless Tobacco Council [STC]) to negotiate a preferred federal warning that could pre-empt an array of troubling warnings in several different states.

#### Rotating cigarette warnings and the addiction issue

Just months before turning to SLT, Congress, the Coalition for Smoking or Health (a coalition of the American Lung Association, the American Cancer Society (ACS), and the American Heart Association (AHA)), and the cigarette industry had worked out a compromise on first-time rotating warnings for cigarettes to go into effect late in 1985 [4] (see Table 1). Without compromise, pro-tobacco forces could have blocked the legislation [29]. The industry might have agreed to the warnings to prevent even tougher warnings (“that cigarettes killed people and were addictive”) if they waited until the following year [29].

#### Federal legislation

In July 1985, the first bill was introduced in the House of Representatives [30]. It proposed three rotating warnings (see Table 1.) A Senate bill was introduced in August with nearly identical warnings. Congressman Waxman chaired hearings on the “Health Effects of Smokeless Tobacco” [30]. In his opening statement, he noted: “Smokeless tobacco is not, as its advertising suggests, a safe alternative to cigarettes” (p. 61). Three supporting legislators followed, each concluding that the public was misled by industry into thinking SLT was a safe or harmless alternative to cigarettes. One also noted that “The American Medical Association has formally declared that ‘snuff dipping and tobacco chewing are hazardous to health, and are not safe alternatives to smoking’” (p. 104) [30].

Sean Marsee’s mother testified that product endorsements by athletes led her son to believe SLT was safe. A researcher presented survey results interpreted as showing “...the students perceive smokeless tobacco as safe...” [30] (p121).

The large survey of over 5000 high school students that the researcher relied on at the hearings was arguably the most persuasive evidence available of what students thought about the risks of SLT (see [31]). In the Waxman hearings, the researcher summarized, “They feel--77 percent of them feel--cigarettes are harmful, where only 40 percent feel smokeless tobacco is harmful. This data suggests [*sic*] the students perceive smokeless tobacco as safe...” (p. 121) [30]. The complete reporting of the results, however, shows a different picture. When asked, “How harmful is dipping/chewing to a person’s health?”, 40% did report “very harmful,” but another 40% said “somewhat harmful,” 15% said “slightly harmful,” and *only 3% reported “not harmful*.” When asked “How harmful is smoking cigarettes to a person’s health?” 77% said “very harmful,” 17% “somewhat harmful,” 3% said “slightly harmful,” and 2% “not harmful.” The clear conclusion, but one not offered in the hearings, is that almost none of the school-age children thought that either SLT or cigarettes was harmless, and, correctly, they thought smoking was more harmful than SLT. The publication reveals another pertinent result: “Can dipping or chewing cause cancer?” Two-thirds (67%) said “yes,” 12% “did not know,” and only 27% said “no.” (The percentages reported add up to 106%, indicating some error in the Table.) To conclude that these youth considered SLT safe was then unsupported by the results in which the majority said it caused cancer and nearly all thought it was harmful.

A survey (*N* = 525) was also released by the Office of the Inspector General in January 1986—before warnings were introduced [32]. Similarly, it found, “Eighty (80) percent of junior high and 92 percent of senior high students acknowledge that dipping and chewing *can be* harmful to a person’s health.” Seventy-nine percent “note the potential risk of oral cancer.” (p. 15) The report also indicated that “a large majority of smokeless users in our sample regard snuff dipping as much safer than smoking and they do not smoke cigarettes now nor do they intend to smoke in the future” [32].

An industry document from October 1985 itemized key provisions of the bills and described a possible, industry-favorable, “alternative bill” [33]. The industry preferred a single warning: “Surgeon General’s Warning: Use of This product is Hazardous To Your Health and May Cause Mouth Disorder. Consult Your Dentist About Any Abnormalities.” A Philip Morris observer reviewed key issues [34-36].

By October 1985, the addiction warning was in the process of being replaced with the NSAC warning [37]. The National Institutes of Health conducted a “Consensus Conference on the Health Implications of Smokeless Tobacco Use” in January and its summary report was included in the Congressional Record before bill passage [38]. Noting that smoking was a major cause of oral cancer, there were conclusions in support of SLT also causing oral cancer and mouth problems, but not for cancer in other sites. “No direct epidemiological data” were available on cardiovascular morbidity or mortality. Likely addiction problems and possible pregnancy and infant health issues were discussed (but would also be problems for smoking). In short, no inference was supported that SLT approached the harmfulness cigarettes. This was also true for the first Surgeon-General’s Report on SLT a few months later [1].

In February 1986, Congressman Waxman maneuvered to get a bill that now included a ban on electronic media advertising approved by the House and sent to the Senate in what was essentially a take-it-or-leave-it gambit [39]. He succeeded [38]. Although state package warnings were pre-empted, there was no specification that the warnings would be legally adequate to prevent liability lawsuits, as had been the case for cigarette warnings [40]. Nevertheless, lawyers thought the required warnings would make liability suits harder to win [41].

While there were repeated assertions in the congressional hearings that SLT was ‘not safe’ or ‘harmless,’ there is only one instance of saying that SLT is not *safer* than cigarettes. In Congressman Synar’s final remarks before passage, he said:

I strongly believe that this warning [on addiction[ is warranted given the state of scientific knowledge on smokeless tobacco. However, in negotiating an overall agreement on this legislation, this label was substituted for one which reads: “Warning: This Product Is Not a Safe Alternative to Cigarettes.” The parties involved in negotiating this agreement felt that such a warning was necessary given the widespread impression that smokeless tobacco use is safer than smoking. [38] (p. H 252)

Table 2 provides an outline of the major factors that influenced both the lack of a warning before 1986 and the creation of warnings in 1986.

**Table 2:** Key factors influencing tobacco control efforts against smokeless tobacco—USA up to 1986

Factor	Before: 1960s–1980	During: 1981–1986
1) Evidence of disease	Smokeless was not judged officially to cause any disease in the USA, while smoking was judged a major cause of serious disease and disability.	Smokeless was judged “not safe”: 1) An NCI study treated as definitive on oral cancer 2) Prominent case of young man viewed as dying from oral cancer from smokeless
2) Use of smokeless	Declining use by mostly older adults in rural areas and some occupations	Rising use in general population and by youth
3) Public perception and industry marketing of smokeless as a *safe* alternative to cigarettes	Marketing of smokeless tobacco with no health warnings, but for cigarettes health warnings and several years no radio/TV marketing	Widespread radio/TV and print marketing of Skoal Bandits® with “Take a pouch instead of a puff” slogan and endorsements by athletes
4) Evidence-based assessment that smokeless is *not safe*, needs a warning label, and other control measures	None	a) Petition to Federal Trade Commission b) Action by several states to require warning labels c) Legislation on rotating warnings, ban of electronic advertising, and other measures
5) Evidence-based assessment that smokeless tobacco is *not safer* than cigarettes	None	None

## Discussion

Both the smokeless and cigarette industries (then separate) wanted to avoid a nicotine/addiction warning. The anti-SLT forces could concede on their preferred nicotine/addiction warning, because cigarettes themselves had not yet been officially declared addictive and such a warning had not been applied to cigarettes only the year before. The agreed-to warnings (on mouth cancer, gum disease, and tooth loss) already expressed that SLT was ‘not safe.’ The industry may have viewed the ‘not safe’ message as akin to their preferred warning that SLT is “hazardous” (see wording above). There is no evidence that any research on consumer reactions was conducted. It was likely viewed by anti-SLT forces as a safe, true message that countered the Skoal Bandits® promotions as had been opposed since 1984 [23, 25]. Given the multiple important issues in play (e.g., the electronic advertising ban), the compromise message might also have seemed a minor issue. An anonymous reviewer of an earlier version of this paper indicated based on information from a key party involved that a cigarette company had insisted on the NSAC warning. As noted above, the cigarette industry disliked the Skoal Bandits® campaign that seemed to ‘denigrate’ cigarettes [21], so indeed it is possible that the cigarette industry desired or even insisted on this warning to replace the addiction warning.

### The evidence-base supported that SLT was ‘not safe,’ not that it was as harmful as cigarettes

This history makes it clear that the only assessment at issue was whether SLT was “not safe” or “harmful.” Congressman Synar’s representation that “...such a warning was necessary given the widespread impression that smokeless tobacco use is safer than smoking” [38] (p. H 252) can be viewed as unjustified by the evidence presented. No determination whatsoever was made of how the harms of SLT compared to those of cigarettes. Perhaps Synar was thinking that both products are addictive, so one is, thus, no safer than the other. Psychological models with their roots in anthropology make it clear, however, that much human thinking is simplistic [42]. For example, the identification of any contamination or pollution can have an all-or-nothing, categorical effect on the perception of a product. This has been referred to as the law of contagion [43]. While toxicologists appreciate risk thresholds and consider dose-response effects of a toxin, the lay public may often simply parse things into ‘safe’ or ‘unsafe’ [44]. Synar’s characterization may be the first example of a particular misinterpretation of the NSAC warning that subsequent research (below) has shown is all too common.

In the several US governmental evaluations of the health effects of SLT over the past three decades (e.g., [1, 14, 45, 46]), the focus has been on identification of harms. Electronic searches of these subsequent reports from the US government finds no quantitative assessments were made on the degree of differential harms from SLT and cigarettes (for example, comparisons of all-cause mortality estimates) [46-48]. ACS researchers who have some of the most definitive US epidemiological datasets have had the capacity for decades to report all-cause mortality comparisons, but have not done so, out of disapproval of using SLT for harm reduction. Nevertheless, the ACS experts do report that “the hazards associated... with [SLT] in this and other studies. .. *are considerably smaller* [emphasis added] than the risks associated with cigarettes” (p. 356) [49].

Direct assessments by others of differential harms also leave no doubt at all that manufactured SLT in North America and Scandinavia is much less harmful than smoking to its users [50–52]. Two basic arguments establish the major differential harms. First, cigarettes are a greater cause of the problems caused by SLT (including oral cancer [53]). Second, cigarettes are also an important cause of major problems that SLT has little to no association with: lung cancer, chronic obstructive lung disease, and other respiratory diseases. These diseases account for the majority of smoking deaths [50]. The AHA has concluded that smoking is a greater cause of heart disease than SLT [54]. Experts estimate that manufactured SLT is likely to be at least 95% less harmful than cigarettes [51, 52].

Finally, in March 2018, FDA Centre for Tobacco Control scientists published a major policy-focused paper [55] that explicitly uses all-cause-mortality estimates for SLT as an example of a meaningful tobacco harm reduction. This indicates acceptance of the conclusions of ACS scientists of “considerably smaller” harms from SLT than cigarettes [49]. In their simulation, they use the age-adjusted, all-cause mortality hazard ratio (HR) estimate for SLT of 1.18. The all-cause mortality HRs for current cigarette smoking were derived for specific age groups for males and females. For example, for males aged 55–64, the HR was 2.79 (95% CI, 2.28, 3.40) and for females aged 55–64 was 2.35 (95% CI, 1.92, 2.87). Overall, this shows a larger mortality risk from cigarettes than SLT [56].

### More recent research shows that the ‘not safe’ message often means ‘just as harmless’ or ‘not safer’

It is easy to imagine why legal departments might like the NSAC message as true and seemingly noncontroversial. But a warning label needs to be judged by its interpretation by recipients [44, 57]. A recent interview study of smokers explored interpretation of the NSAC message [6]. The results show that the warning is conveying other than a simple “not a harmless product” message. The authors wrote: “Several thought that it suggested a message that although some might think SLT is less harmful than cigarettes, it is really not...Several thought it gave the impression that SLT is just as harmful as cigarettes.” (p. 673). Another report found that participants actually pointed to the NSAC message as supporting evidence that SLT and cigarettes were equally harmful [7].

### Implications for warnings about emerging ‘not safe, but safer’ tobacco/nicotine products

Research using a NSAC warning on vaping products (e-cigarettes) reveals a similar problem of misinterpretation [8]. This focus-group study revealed complex reactions to the message. For those who thought it an effective warning: “Many...thought it could be a strong warning because people generally think e-cigarettes are safer than cigarettes, and that this statement countered those perceptions.”(p. 8). Respondents thought the statement suggested that there would be no health benefit in switching from cigarettes to e-cigarettes. (p 8) One respondent said “...obviously it’s saying that it’s not a safer alternative to cigarettes, so why go to it” (p. 8). Another respondent said “So this kind of I think gives the impression that it’s not safer than cigarettes” (p 8).

### International support for the ‘not a safe alternative’ warning

Although some jurisdictions have banned smokeless tobacco but not cigarettes (e.g., Australia and the European Union outside of Sweden), where available appropriate warnings should be employed. The World Health Organization (WHO) has accepted that the public has a right to accurate information on the harms of tobacco and that “countries have a legal obligation to provide it” [5] (p. 21). In 1988, WHO Technical Report [58] recommends the NSAC warning be included in rotating warnings for SLT. By 2000, Canada required rotating warnings that included the NSAC warning [59].

### Limitations

The tobacco industry documents cannot be assumed to represent all discussions and perspectives that may have been at issue surrounding the warning labels. Alternative interpretations of the documents, media reports, and hearing testimony may be possible. For complex historical issues, any one person’s account of the explanations for events, even from a witness or participant may not be an accurate rendering of the matter.

## Conclusions

The warning that “Smokeless tobacco is not a safe alternative to cigarettes” arose as a compromise to prevent an addiction warning and an imminent diversity of state warnings. The cigarette industry and the separate SLT industry would have equally wanted to avoid an addiction warning, and the cigarette companies may have insisted on the NSAC warning as also in their interest. Public health forces would likely have equally welcomed the NSAC message. The evidence presented supported the conclusion that there were some harms caused by smokeless tobacco and no assessment was made of harms relative to cigarettes. Despite contemporary expert interpretations to the contrary, surveys prior to the warning indicated that the public generally did not view smokeless tobacco as safe and they did generally report smokeless as less harmful than cigarettes.

Though many countries have banned smokeless tobacco (but not cigarettes), where smokeless is legally available accurate information on the nature of harms and differential harms need to be developed. The NSAC warning persists unchanged to this day and might similarly be applied to other less harmful tobacco/nicotine products (e.g., vaping, e-cigarettes), despite research indicating significant problems with comprehension (e.g., [6]). The popular use of similar “not harmless” and “no safe tobacco product” messages (e.g., [60, 61]) may be also susceptible to misinterpretation, especially in the absence of information expressing differential product harms. Although this would not have been appreciated by public health authorities in the 1980s at the initiation of the NSAC warning, it has emerged subsequently that the official warning informs many people that SLT is, in effect, *not a safer* alternative to cigarettes and is deceptive and inconsistent with communication standards [62, 63]. Recent research shows that few of the public now think that SLT is less harmful than cigarettes [64]. Systematic, health communication research is needed to replace this type of warning with one that is ethically responsible and based on scientific evidence rather than possibly well-intended, but unethical manipulation of the public [62, 64–68]. An assessment of whether a product is ‘not safe’ is not the same as an assessment of it being ‘not safer’ than cigarettes. In tobacco harm reduction, well-tested messages are needed to communicate the magnitude of differential product harms.

## Abbreviations

ACS: American Cancer Society
CDC: United States Centers for Disease Control and Prevention
FDA: United States Food and Drug Administration
FTC: United States Federal Trade Commission
HR: Hazard ratio
NCI: United States National Cancer Institute
NSAC: Not a safe alternative to cigarettes
PCHRG: Public Citizen Health Research Group
SLT: Smokeless tobacco
US: United States of America
WHO: World Health Organization
